# Effect of interleukin-6 polymorphism on risk of preterm birth within population strata: a meta-analysis

**DOI:** 10.1186/1471-2156-14-30

**Published:** 2013-04-25

**Authors:** Wilfred Wu, Erin A S Clark, Gregory J Stoddard, W Scott Watkins, M Sean Esplin, Tracy A Manuck, Jinchuan Xing, Michael W Varner, Lynn B Jorde

**Affiliations:** 1 Department of Human Genetics, University of Utah School of Medicine, Salt Lake City, UT 84112, USA; 2 Department of Obstetrics and Gynecology, University of Utah School of Medicine, Salt Lake City, UT, 84112, USA; 3 Study Design and Biostatistics Center, University of Utah School of Medicine, Salt Lake City, UT, 84112, USA; 4 Department of Genetics, Rutgers, The State University of New Jersey, Piscataway, NJ, 08854, USA

**Keywords:** Preterm birth, Preterm delivery, Premature birth, Premature delivery, Genetic polymorphism, Genetic variant, Single nucleotide polymorphism, SNP, Interleukin-6, Cytokine, Population structure, HapMap project, Phenotype heterogeneity

## Abstract

**Background:**

Because of the role of inflammation in preterm birth (PTB), polymorphisms in and near the *interleukin-6* gene (*IL6*) have been association study targets. Several previous studies have assessed the association between PTB and a single nucleotide polymorphism (SNP), rs1800795, located in the *IL6* gene promoter region. Their results have been inconsistent and SNP frequencies have varied strikingly among different populations. We therefore conducted a meta-analysis with subgroup analysis by population strata to: (1) reduce the confounding effect of population structure, (2) increase sample size and statistical power, and (3) elucidate the association between rs1800975 and PTB.

**Results:**

We reviewed all published papers for PTB phenotype and SNP rs1800795 genotype. Maternal genotype and fetal genotype were analyzed separately and the analyses were stratified by population. The PTB phenotype was defined as gestational age (GA) < 37 weeks, but results from earlier GA were selected when available. All studies were compared by genotype (CC versus CG+GG), based on functional studies.

For the maternal genotype analysis, 1,165 PTBs and 3,830 term controls were evaluated. Populations were stratified into women of European descent (for whom the most data were available) and women of heterogeneous origin or admixed populations. All ancestry was self-reported. Women of European descent had a summary odds ratio (OR) of 0.68, (95% confidence interval (CI) 0.51 – 0.91), indicating that the CC genotype is protective against PTB. The result for non-European women was not statistically significant (OR 1.01, 95% CI 0.59 - 1.75). For the fetal genotype analysis, four studies were included; there was no significant association with PTB (OR 0.98, 95% CI 0.72 - 1.33). Sensitivity analysis showed that preterm premature rupture of membrane (PPROM) may be a confounding factor contributing to phenotype heterogeneity.

**Conclusions:**

*IL6* SNP rs1800795 genotype CC is protective against PTB in women of European descent. It is not significant in other heterogeneous or admixed populations, or in fetal genotype analysis.

Population structure is an important confounding factor that should be controlled for in studies of PTB.

## Background

Preterm birth (PTB) is defined as birth before 37 completed weeks’ gestation. Prematurity is the leading cause of neonatal mortality in newborns without congenital anomalies or chromosomal abnormalities [1,2]. It is also associated with a broad spectrum of lifelong morbidity in survivors, including neurodevelopmental delay, cerebral palsy, blindness, deafness, and chronic lung disease [3,4]. Despite efforts to reduce preterm birth, the rate has remained relatively stable over the last few decades and was 11.7% in the United States in 2011 [1,5]. Although PTB is a pressing public health issue, the incomplete understanding of genetic and environmental risk factors has inhibited development of effective prevention and treatment strategies.

The etiology of PTB is complex and multifactorial [6-9]. There is compelling evidence that both maternal and fetal genomes contribute to risk [10-12], and PTB prevalence varies among population groups [13-17]. African-American ancestry is consistently associated with an increased risk of preterm birth even after adjusting for epidemiologic risk factors, such as income, education, lack of prenatal care, and other socioeconomic factors [6,18,19]. The heritability of PTB, based on twin studies, is estimated to be approximately 30% [20-22].

Despite strong evidence of a genetic component, consistent identification of risk loci has been challenging. A promising candidate gene has been *interleukin-6* (*IL6*). This gene encodes the pro-inflammatory cytokine interleukin-6 (IL-6), which is involved in the regulation of innate immunity. Several studies have shown that PTB is associated with increased concentration of IL-6 in maternal serum, cervicovaginal secretions and amniotic fluid [23-25]. Many *IL6* polymorphisms have been assessed for association with preterm birth [26]. A single nucleotide polymorphism (SNP), rs1800795, usually referred to as *IL6* -174 (from the transcription start site) or -237 (from the translation start site), is located within the *IL6* promoter region [27,28] and is one of the most thoroughly studied *IL6* variants. This SNP is located in the segment of the *IL6* promoter that is crucial for transcriptional induction with viruses and other cytokines [27,28]. Expression studies of IL-6 with allelic variants of the rs1800795 polymorphism have produced different results in different tissues [29-31]. Nevertheless, studies using HeLa cell lines showed that the derived C-allele is associated with a significantly lower level of *IL6* expression (0.624-fold lower) [32]. In addition, adults with the CC genotype have significant lower plasma concentrations of IL-6, compared with adults with the GG or GC genotypes (1.63 pg/mL v.s. 2.74 or 2.64 pg/mL) [32].

Studies of the association between the *IL6* rs1800795 polymorphism and PTB have yielded inconsistent results. Simhan et al. (2003) reported that the CC genotype is protective for PTB compared to the CG and GG genotypes [33]. However, others [34-44] have shown no significant association between *IL6* and PTB. Hapmap data show that the C allele has a frequency of 0.53 in the CEU population (Utah residents with ancestry from northern and western Europe), but it is absent in the YRI (Yoruba in Ibadan, Nigeria), CHB (Han Chinese in Beijing, China) and JPT (Japanese in Tokyo, Japan) populations [45]. Because previous studies have been limited by small sample size and by potential population stratification, we hypothesized that the inconsistent results for rs1800795 are, at least in part, explained by differences in population structure and admixture. Here, we carry out a meta-analysis to systematically review the association of rs1800795 with spontaneous PTB, and to specifically explore the SNP association within population strata.

## Results

### Included studies

Thirty-three articles were identified from the initial keywords search. After review, 21 were excluded (see Additional file 1). Table 1a and 1b summarize the characteristics of the studies included in the meta-analysis, including study populations, whether or not preterm premature rupture of membrane (PPROM) was excluded, cutoff for the cases and controls, and sample sizes (Table 1a and 1b). For maternal genotype analysis, a total of ten studies were included. This resulted in 1,165 PTB cases and 3,830 term controls, more than tripling the case sample size of any individual study (Table 1a). For the fetal analysis, only four studies could be included, with 785 PTB cases and 882 term controls (Table 1b).

**Table 1:** Characteristics of the included studies


**(a). Characteristics of the studies included for maternal genotype analysis**								
**Author**	**Year**	**Country**	**Populations**	**PPROM Excluded?**	**PTB cases group criteria**	**Case sample size**	**Control group criteria**	**Control sample size**
**Women of European descent**								
Annells et al	2004	Australia	White	No	GA<35	202	GA≥37	185
Hartel et al	2004	Germany	White	No	GA<37 and VLBW	365	GA≥37	281
Hollegaard et al	2008	Denmark	White	No	GA<37	62	GA≥37	55
Menon et al	2005	TN & PA, USA	White	No	GA<36	101	GA≥37	326
Simhan et al	2003	PA. USA	White	Yes	GA<34	39	GA≥37	110
Stonek et al	2008	Austria	White	No	GA<34	21	GA≥37	1367
**Heterogeneous population**								
Gomez et al	2010	PA, USA	African American, and others	No	GA<37	60	GA≥37	636
Harper et al	2011	USA	White, African American, Asian, and Others	No	GA<28	33	GA≥37	549
Moura et al	2009	Brazil	Mulatto	No	GA<37	111	GA≥37	94
Moura et al	2009	Brazil	Mulatto and White	No	GA<37	80	GA≥37	101
Simhan et al	2003	PA. USA	African American	Yes	GA<34	12	GA≥37	46
Speer et al	2006	IL, USA	White, African American, and Others	No	GA<35	79	GA≥37	80
**(b). Characteristics of the studies included for fetal genotype analysis**								
**Women of European descent**								
Hartel et al	2004	Germany	White	No	GA<37 and VLBW	606	GA≥37	491
**Heterogeneous population**								
Pereyra et al	2012	Uruguay	Uruguayan	No	GA<37	53	GA≥37	56
Speer et al	2006	IL, USA	White, African American, and Others	No	GA<35	78	GA≥37	78
Velez et al	2007	TN & PA, USA	African American	Yes	GA<36	48	GA≥37	257

Each study was stratified by population into (1) European ancestry or (2) heterogeneous population – either the studied samples were from admixed populations or contained several different populations and could not be stratified based on available information. All ancestry was self-reported. None of the studies used ancestry-informative markers to determine the ancestry of the samples.

Among all the included studies, only one in the maternal group [33] and one in the fetal group [44] excluded PPROM (Table 1a and 1b). Several studies had a gestational age (GA) cut-offs earlier than 37 weeks: Harper et al. [36] at 28 weeks, Simhan et al. [33] and Stonek et al. [43] at 34 weeks. Hartel et al. [37] used a GA cut-off of 37 weeks and very low birth weight as the PTB case criteria, thereby selecting a more severe phenotype.

### Association of genotype and phenotype

**Figure 1:**
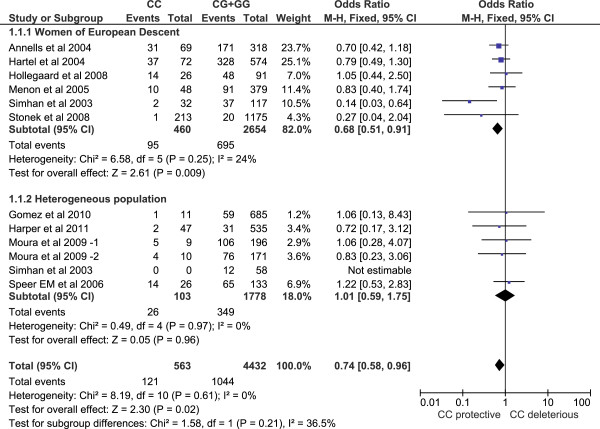
**Forest plot for maternal genotype analysis. **The count for genotypes, weight, OR, 95% confidence interval for each study; summary for each subgroup population; and heterogeneity test statistics are shown. Each square represents an OR for each specific study, and the area is proportional to the weight. Each horizontal line shows the 95% CI for each study. The diamonds are the summary OR for each subgroup population or for the total. The CC genotype is significantly protective against PTB in women of European descent (OR=0.68, 95% CI 0.51 – 0.91). It is not significant in heterogeneous populations.

**Figure 2:**
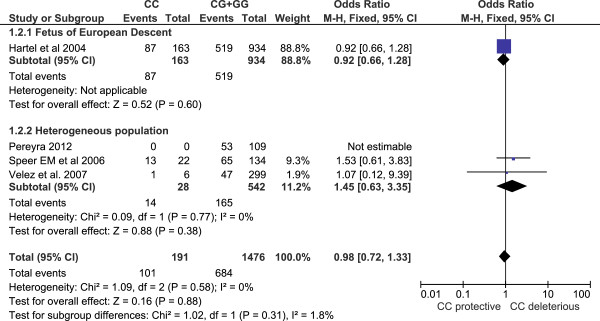
**Forest plot for fetal genotype analysis. **The count for genotypes, weight, odds ratio, 95% confidence interval for each study; summary for each subgroup population; and heterogeneity test statistics are shown. Each square represents the odds ratio for each specific study, and the area is proportional to the weight. Each horizontal line shows the 95% confidence interval for each study. The diamonds are the summary odds ratio for each subgroup population or for the total. The association between rs1800795 and PTB is not significant for fetuses of European descent, or for those of heterogeneous populations.

Both Cochrane's Q test and the I-square statistic suggest that there is no evidence of heterogeneity across all studies or for each subgroup (Figures 1 and 2), for maternal or fetal genotype analysis. Thus, a Mantel–Haenszel fixed-effect model [46] was employed to pool all studies for summary OR estimation.

For the maternal genotype analysis, the overall OR is 0.74 for genotype CC versus CG+GG (95% CI 0.58 – 0.96), demonstrating a significant protective effect of the CC genotype. In subgroup analysis, women of European descent have an OR of 0.68 (95% CI 0.51 - 0.91), but the association is not significant in other populations (OR 1.01, 95% CI 0.59 – 1.75) (Figure 1 Forest plot).

For the fetal genotype analysis, the overall OR for genotype CC versus CG+GG is 0.98 (95% CI 0.72 – 1.33). Although genotype CC showed a trend toward a protective effect in infants of European descent, the result is not significant (Figure 2 Forest plot).

### Bias assessment

For maternal genotype sensitivity analysis, the overall OR after exclusion of any individual study showed no change of the trend - ranging from 0.71 to 0.81 - indicating a robust protective effect of the CC genotype against PTB. In the European descent subgroup, the sensitivity analysis was still stable, with summary ORs ranging from 0.63 to 0.76. However, exclusion of one study [33] produced a summary OR with borderline significance (OR 0.76, 95% CI 0.57 – 1.03) (Table 2a). Interestingly, this was the only maternal study that excluded PPROM (Table 1a). This result suggests that PPROM may thus be a confounding factor and deserves separate analysis to reduce phenotypic heterogeneity. Unfortunately, we had insufficient data to stratify by the presence or absence of PPROM. Fetal genotype sensitivity analysis also showed no change in the trend (Table 2b).

**Table 2:** Sensitivity analysis


**(a). Maternal genotype sensitivity analysis**		
**Study omitted**	**Overall OR**	**Subgroup OR**
**Women of European descent**		
Annells et al 2004	0.76 [0.57, 1.01]	0.68 [0.48, 0.95]
Hartel et al 2004	0.73 [0.54, 0.98]	0.63 [0.45, 0.90]
Hollegaard et al 2008	0.72 [0.55, 0.94]	0.65 [0.48, 0.88]
Menon et al 2005	0.73 [0.56, 0.96]	0.66 [0.48, 0.90]
Simhan et al 2003	0.81 [0.63, 1.05]	0.76 [0.57, 1.03]
Stonek et al 2008	0.76 [0.59, 0.99]	0.71 [0.53, 0.94]
**Heterogeneous population**		
Gomez et al 2010	0.74 [0.57, 0.95]	1.01 [0.57, 1.78]
Harper et al 2011	0.74 [0.57, 0.96]	1.08 [0.60, 1.96]
Moura et al 2009 -1	0.73 [0.57, 0.95]	1.00 [0.55, 1.83]
Moura et al 2009 -2	0.74 [0.57, 0.96]	1.06 [0.58, 1.93]
Simhan et al 2003	n/a	n/a
Speer EM et al 2006	0.71 [0.54, 0.92]	0.88 [0.43, 1.83]
**(b). Fetal genotype sensitivity analysis**		
**Study omitted**	**Overall OR**	**Subgroup OR**
**Women of European descent**		
Hartel et al 2004	1.45 [0.63, 3.35]	n/a
**Heterogeneous population**		
Pereyra et al 2012	n/a	n/a
Speer et al 2006	0.92 [0.66, 1.28]	1.07 [0.12, 9.39]
Velez et al 2007	0.97 [0.71, 1.33]	1.53 [0.61, 3.83]

**Figure 3:**
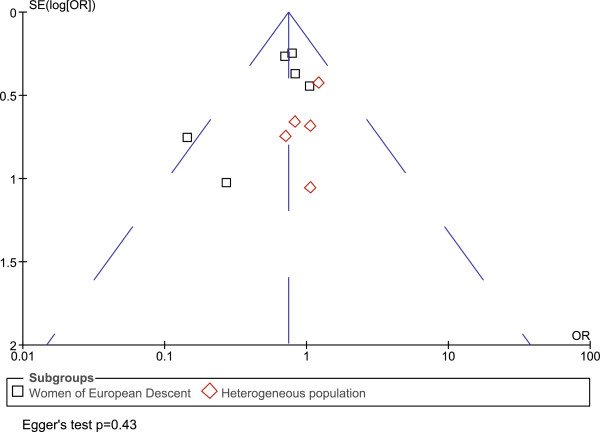
**Funnel plot for maternal genotype analysis. **Funnel plot was showing OR versus standard error (SE). Egger’s test p value is also shown. Each square or diamond represents a study. Squares and diamonds represent different subgroup populations. There is no evidence of publication bias.

**Figure 4:**
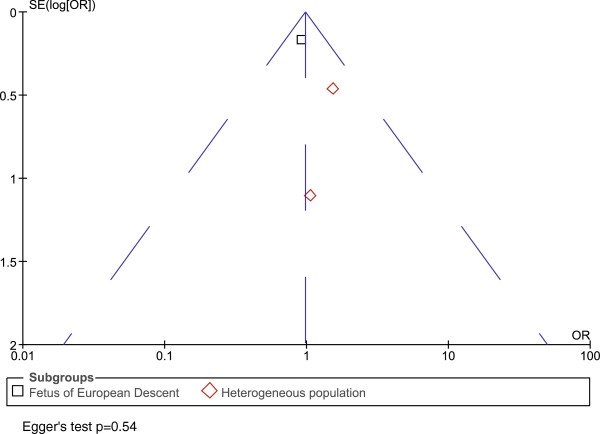
**Funnel plot for fetal genotype analysis. **Funnel plot showing OR versus standard error (SE). Egger’s test p value is also shown. Each square or diamond represents a study. Squares and diamonds represent different subgroup populations. There is no evidence of publication bias.

Funnel plots were symmetrical for both the maternal and fetal analyses, indicating no publication bias. Egger's test [47] also showed no evidence of publication bias (p=0.43 for maternal genotype analysis, p=0.54 for infant genotypes) (Figures 3 and 4 Funnel plots).

## Discussion

Population heterogeneity may help to explain why results among genetic studies of rs1800795 and PTB association were inconsistent. Indeed, HapMap data showed that the frequency of the derived C allele of rs1800795 is 0 in the YRI, CHB, and JPT populations, while it is 0.53 in the CEU population [45]. In other population samples, the C allele frequency is consistently high in people of European descent, ranging from 0.35 in Tuscans from Italy to 0.65 in a European-American sample ascertained for coronary artery disease. In contrast, the C allele frequency is consistently low in East Asian (highest in Chinese in Metropolitan Denver, Colorado, 0.006) and African (highest in Maasai in Kinyawa, 0.049) populations. The frequency in admixed populations varies. The Programs for Genomic Applications' African-American panel reported a frequency of 0, a population with African ancestry in Southwest USA had a frequency of 0.092, while a population with Mexican ancestry in Los Angeles had a frequency of 0.16 [48]. The allele frequency difference of 0.53 between people of European descent and populations in other continents falls within the 5% tail of continental allele frequency differences for a large panel of common SNPs (see Additional file 2) and is thus statistically significant. This variation suggests that population heterogeneity could have strong effects for this polymorphism. After stratification by population, our subgroup analysis showed that the rs1800795 CC genotype is significantly protective against PTB in women of European descent, but not in other heterogeneous populations (Figure 1). In the latter group of populations, ancestral diversity is likely to obscure any potential genotype-phenotype association, emphasizing the importance of addressing underlying population structure in genetic studies.

A regression analysis reveals a negative relationship between OR and CC genotype frequency (see Additional file 3). The result is significant in maternal studies. This matches the finding that in European populations, which have high CC genotype frequencies, the CC genotype has a protective effect against PTB; while in ethnically heterogeneous populations, which have low or zero CC genotype frequencies, no protective effect could be observed.

In all these studies, all ancestry was self-reported. Unfortunately, genetic admixture data confirming self-reported ancestry are not available. However, previous studies have demonstrated nearly 100% concordance between self-reported race/ethnicity and specific genetic ancestry markers among pregnant women enrolled in clinical studies [18]. Although we cannot exclude the possibility of misclassification of some women, we infer a similar high rate of concordance between self-reported race/ethnicity and genotyped race/ethnicity among women enrolled in this meta-analysis.

It is intriguing that rs1800795 has such large frequency differences across different populations. In addition to its association with PTB, this SNP has been shown to be associated with many other diseases, such as juvenile rheumatoid arthritis [32], susceptibility to Kaposi sarcoma [49], metabolic syndrome [50-54], and inflammatory bowel disease [55]. However, there is no strong evidence of natural selection near rs1800795 [56-58].

Methodological differences may have contributed to the inconsistent results among prior studies. Most studies compared CC vs. CG+GG, but some reported GG vs. CG+CC and others compared individual alleles rather than genotypes. Because of the evidence that CC genotypes cause significantly lower serum IL-6 concentrations than do the CG or GG genotypes [32], we uniformly meta-analyzed CC versus CG+GG for all studies, providing a consistent approach to the genotype-phenotype correlation.

Another goal of our meta-analysis was to increase statistical power by increasing the sample size. For women of European descent, we pooled 790 PTB cases and 2,324 controls, which significantly increased the sample size relative to any previous individual study. With this sample size, we have 97.72% power to detect an OR of 0.68 at the 0.05 significance level. Under the same conditions, the single study with the largest case sample size [37] had only 51.38% power.

The purpose of this meta-analysis is to pool peer-reviewed published studies that qualify our inclusion criteria (see Additional file 1). Even though there is no evidence of publication bias (Figures 3 and 4), a potential limitation of our study is that some findings of no association between rs1800795 and PTB may not have been reported in the literature and therefore could not be included in our meta-analysis.

In addition to population structure, phenotypic heterogeneity is another problem that may undermine studies of any complex diseases. In this meta-analysis, we stratified by population, included the data from earlier GA cutoffs, and selected earlier PTB cases. Therefore, our conclusion that the CC genotype of rs1800795 in the *IL6* promoter is protective against PTB is limited to European women with early PTB. Our sensitivity test showed that PPROM may be a confounding factor. Besides early PTB and PPROM, further refinement of PTB phenotype, such as a sub-classification by placenta abruption, cervical insufficiency, and other factors, may also help to reduce phenotype heterogeneity.

## Conclusions

In summary, we performed a meta-analysis of the association between PTB and a polymorphism located in the *IL6* promoter region, SNP rs1800795. We specifically stratified the analysis by population subgroup and selected for early PTB. We found the derived CC genotype is protective against PTB in women of European ancestry. No significant associations were found for non-European samples, in whom the CC genotype frequency is low. PPROM may be a confounding factor contributing to phenotype heterogeneity and deserves further investigation.

## Methods

### Data sources

A literature search was conducted in PubMed (U.S. national Library of Medicine, Jan 1966-April 2012) to identify studies of the association between *IL6* rs1800795 and PTB. Keywords included: (“interleukin 6 polymorphism”, or “interleukin 6 variant”, or “interleukin 6 genotype”), AND (“preterm birth” OR “preterm delivery”). The “AND” operator was used to combine these terms in varying combinations. No search software was used. After these studies were retrieved, they were individually reviewed. Bibliographies of all articles retrieved were further reviewed for potentially eligible studies (see Additional file 1).

### Study selection and data extraction

We included human studies with: (1) a genotype of *IL6* SNP rs1800795 (also referred as *IL6* -174 or -237) and (2) a collection of affected PTB cases and unaffected controls. Two authors (W.W. and E.A.S.C) independently searched and reviewed the articles. If studies only presented summary data, we contacted the authors for genotype counts and population stratification as needed (see Additional file 1). Publications were excluded if the rs1800795 genotype distribution for cases and controls could not be determined, or if the control group was reported to deviate significantly from Hardy-Weinberg equilibrium. Non-English language papers were also excluded. When two or more articles were published by the same group of authors, we evaluated the studies for evidence of overlapping samples. If the paper, or author correspondence, suggested overlapping cohorts, we included only the first study for meta-analysis. The study population geographic origins, criteria for PTB diagnosis, occurrence of PPROM, sample source (maternal or fetal), and genotype count for affected and unaffected individuals were extracted.

### Selection of outcomes

All studies of PTB with GA <37 weeks were eligible for inclusion. If multiple GA cut-offs <37 weeks were reported, we selected the cut-off that was one level earlier than 37 weeks in order to ensure an accurate phenotype and to reduce phenotype heterogeneity. This approach allows us to reduce classification error for PTB occurring near GA=37 weeks, and tends to select for a more severe phenotype (earlier PTB), further helping to reduce phenotype heterogeneity.

PPROM is a distinct subset of PTB and is frequently excluded in PTB studies [26]. Exclusion of PPROM would further reduce phenotype heterogeneity; however, among all *IL6*-PTB association studies, only two excluded PPROM [33,44] (Table 1a and 1b). Therefore, study selection was not predicated on exclusion of PPROM.

### Statistical analysis

The meta-analysis compared the CC versus CG+GG genotypes, based on prior functional studies that suggest a similar phenotype for the latter two genotypes [32]. Maternal and fetal genotypes were analyzed separately. Heterogeneity was assessed by Cochrane’s Q test of heterogeneity and the I-square statistic [59,60]. If there was no significant heterogeneity, a Mantel–Haenszel fixed-effect model [46] was employed to calculate the pooled odds ratio (OR) and 95% confidence interval (CI). Otherwise, the random-effects model was used. Subgroup analysis was performed to test for the effects of population stratification. Sensitivity analysis was performed by omitting one publication at a time. Funnel plots and Egger's test [47] were used to assess publication bias. The analysis was carried out using RevMan 5.0 [61], and STATA 11 [62]. This article was prepared based on the guideline of “meta-analyses of observational studies” (MOOSE) [63]. A MOOSE checklist is shown in Additional file 4 (see Additional file 4).

## Abbreviations

IL6: *Interleukin-6* (gene); IL-6: Interleukin-6 (protein); PTB: Preterm birth; GA: Gestational age; OR: Odds ratio; CI: Confidence interval; PPROM: Preterm premature rupture of membrane; HWE: Hardy-Weinberg Equilibrium; MOOSE: Meta-analyses of observational studies.

## Competing interests

None of the authors reports any competing interests relative to the work presented in this manuscript.

## Authors’ contributions

WW conceived of and designed the study. EASC, GS, WSW, MSE, TAM, JX, MV, and LBJ provided input to the study design. WW, EASC, GS, and WSW conducted the analysis. WW and EASC drafted the manuscript. GS, WSW, MSE, TAM, JX, MV, and LJ provided critical review of the manuscript. MV and LBJ oversaw the analysis. All authors read and approved the final manuscript.

## Supplementary Material

Additional file 1 All the included and excluded papers and the reasons of inclusion or exclusion. Click here for file

Additional file 2 Distribution of SNPs allele frequency differences for three continental populations. Click here for file

Additional file 3 Meta-regression analysis for (a) maternal genotype studies, (b) fetal genotype studies. Click here for file

Additional file 4 MOOSE checklist. Click here for file
