# Supplementary material for: Effect of interleukin-6 polymorphism on risk of preterm birth within population strata: a meta-analysis
Source: BMC Genet. 2013 Apr 25;14:30. doi: 10.1186/1471-2156-14-30 (PMC3639799; doi:10.1186/1471-2156-14-30)
Supplement: Additional file 1 — All the included and excluded papers and the reasons of inclusion or exclusion. [file 1471-2156-14-30-S1.pdf]

Additional file 1. All the included and excluded papers and the reasons of inclusion or exclusion

Author	Year	Journal	Human	Inclusion Criteria				Notes
				PTB phenotype	rs1800795 tested	English	HWE tested	
**(a) Included papers**								
Pereyra et al	2012	BMC Research Note	Yes	Yes	Yes	Yes	Yes	
Harper M et al	2011	Obstet Gynecol	Yes	Yes	Yes	Yes	Yes	Contacted the author for the detail genotype count and population stratification.<br>Got the numbers but no population stratification.
Gomez LM et al	2010	Am J Obstet Gynecol	Yes	Yes	Yes	Yes	Yes	Contacted the author for population stratification, but no data available.
Moura E et al	2009	J Reprod Immunol	Yes	Yes	Yes	Yes	Yes	Contacted the author for population stratification, but no data available.
Hollegaard MV et al	2008	Acta Obstet Gynecol Scand	Yes	Yes	Yes	Yes	Yes	Contacted the author for the genotype count and got the data.
Stonek F et al	2008	Am J Reprod Immunol.	Yes	Yes	Yes	Yes	Yes	
Velez DR et al	2007	Ann Hum Genet	Yes	Yes	Yes	Yes	Yes	Contacted the author for the genotype count and got the data.<br>Included the subgroups that are in HWE.
Speer EM et al	2006	Hum Immunol.	Yes	Yes	Yes	Yes	Yes	Contacted the author for population stratification, but no data available.
Menon R et al.	2006	Am J Obstet Gynecol	Yes	Yes	Yes	Yes	Yes	
Annells MF et al.	2004	Am J Obstet Gynecol.	Yes	Yes	Yes	Yes	Yes	
Härtel Ch et al.	2004	Mol Hum Reprod.	Yes	Yes	Yes	Yes	Yes	
Simhan HN et al.	2003	Am J Obstet Gynecol	Yes	Yes	Yes	Yes	Yes	
**(b) Excluded papers**								
Resch B et al	2010	Ann Neurol.	Yes	No				Phenotype studied is chorioamnionitis and cerebral palsy.
Bitner A et a	2010	Arch Med Sci	Yes	No				An exclusively PPROM study.
Romero R et al	2010	Am J Obstet Gynecol	Yes	Yes	No			Did not test rs1800795.
Simhan HN	2010	Am J Obstet Gynecol	Yes	No	No			An editorial comments for Romero''s paper.
Resch B et al	2010	J Perinatol	Yes	No				Phenotype studied is chorioamnionitis and cystic periventricular leucomalacia.
Kalinka J et al	2009	Ginekol Pol	Yes	Yes	Yes	No		An article in Polish.
Sata F et al	2009	Mol Hum Reprod	Yes	Yes	No			Did not test rs1800795.
Reiman M et al	2009	Pediatr Res.	Yes	No				Phenotype studied is the volume of gray matter in preterm infant.
Reiman M et al	2008	J Pediatr	Yes	No				Phenotype studied is chorioamnionitis and neonatal infections in preterm infants.
Fortunato SJ et al	2008	Am J Obstet Gynecol	Yes	Yes	No			Did not test rs1800795.

Velez DR et al	2008	Hum Mol Genet.	Yes	Yes	Yes	Yes	Yes	Missing data. Contacted the author for the genotype count, but no data available.
Göpel W et al.	2006	Genes Immun	Yes	No				Phenotype studied is sepsis susceptibility and intraventricular hemorrhage in preterm infants.
Treszl A et al.	2006	Front Biosci	Yes	No				Phenotype studied is necrotizing enterocolitis.
Mattar et al.	2006	J Reprod Med	Yes	Yes	Yes	Yes	No	Missing data for HWE evaluation. Contacted the author for the genotype count, but no data available.
Engel et al.	2005	Epidemiology	Yes	Yes	Yes	Yes	Yes	Missing data. Contacted the author for the genotype count, but no data available.
Jamie WE et al.	2005	Am J Obstet Gynecol.	Yes	No				Symptomatic study group: Exclusively to woman who experienced uterine contractions between 23 and 32 weeks.
Bokodi G et al.	2005	Eur Cytokine Netw	Yes	No				Phenotype studied is ventilation characteristics in preterm baby.
Vásárhelyi B et al.	2005	Pediatr Nephrol.	Yes	No				Phenotype studied is acute renal failure in preterm baby.
Harding DR et al.	2004	Pediatrics	Yes	No				Phenotype studied is neurological and development severity for preterm infant.
Treszl A et al.	2003	J Pediatr Surg	Yes	No				Phenotype studied is necrotizing enterocolitis in preterm infant.
Yoshimura K et al.	2003	J Soc Gynecol Investig.	No					Murine infection model experiment.

- Inclusion criteria were applied from left to right. The article is excluded when the first “No” occurred.
