# Supplementary material for: Effect of interleukin-6 polymorphism on risk of preterm birth within population strata: a meta-analysis
Source: BMC Genet. 2013 Apr 25;14:30. doi: 10.1186/1471-2156-14-30 (PMC3639799; doi:10.1186/1471-2156-14-30)
Supplement: Additional file 2 — Distribution of SNPs allele frequency differences for three continental populations. [file 1471-2156-14-30-S2.pdf]

Additional file 2. Distribution of SNPs allele frequency differences for three continental populations

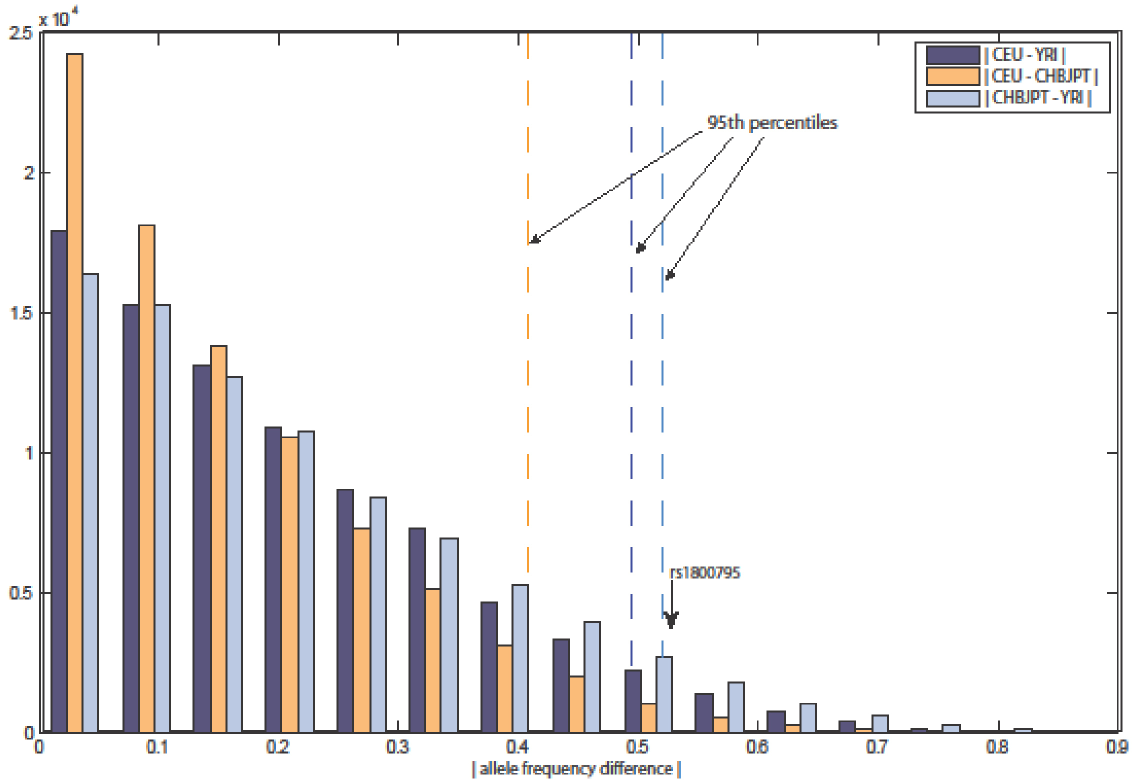

Starting with 815,377 SNPs from the Affymetrix 6.0 chip, SNPs were filtered for linkage disequilibrium ($r^2 \leq 0.2$) leaving 134,288 SNPs, then filtered for SNPs with a minor allele frequency > 0.05 in each of the 3 populations yielding 86,255 common unlinked SNPs. The absolute value of the difference in allele frequency between each of the major population groups was calculated for each SNP. The X axis shows the absolute value of the allele frequency difference. The Y axis is the count of comparisons. Each color represents the pair-wise comparison between two population groups. Dashed lines show the 95% percentile for each distribution.
