# Supplementary material for: Effect of interleukin-6 polymorphism on risk of preterm birth within population strata: a meta-analysis
Source: BMC Genet. 2013 Apr 25;14:30. doi: 10.1186/1471-2156-14-30 (PMC3639799; doi:10.1186/1471-2156-14-30)
Supplement: Additional file 3 — Meta-regression analysis for (a) maternal genotype studies, (b) fetal genotype studies. [file 1471-2156-14-30-S3.pdf]

Additional file 3: Meta-regression analysis for (a) maternal genotype studies, (b) fetal genotype studies

(a) Meta-regression analysis for maternal genotype studies

Author	OR	CC frequency	Sample size
**Women of European descent**			
Annells et al	0.70	0.18	387
Hartel et al	0.79	0.11	646
Hollegaard MV et al	1.05	0.22	117
Menon et al	0.83	0.11	427
Simhan et al	0.14	0.21	149
Stonek F et al	0.27	0.15	1388
**Heterogeneous population**			
Gomez LM et al	1.06	0.02	696
Harper et al	0.72	0.08	582
Moura E et al	1.06	0.04	205
Moura E et al	0.83	0.06	181
Simhan et al	n/a	0.00	58
Speer EM et al	1.22	0.16	159

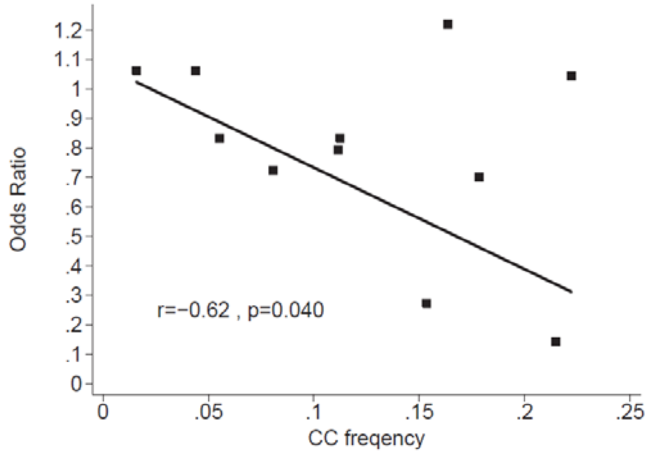

(b) Meta-regression analysis for fetal genotype studies

Author	OR	CC frequency	Sample size
**Women of European descent**			
Hartel et al	0.92	0.15	1097
**Heterogeneous population**			
Pereyra et al	n/a	0.00	109
Speer EM et al	1.53	0.14	156
Velez et al	1.07	0.02	305

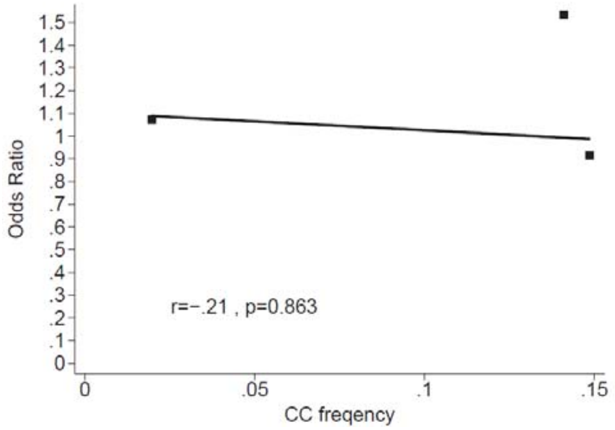

The odds ratio (OR), CC genotype frequency at rs1800795, and total sample size are shown for each study in the tables. Each study was represented as a square on the scatter plot with OR on Y axis, and CC frequency on X axis. Regression analysis was performed by: OR as a dependent variable, CC frequency as an independent variable, and each study was scaled by its sample size while the total number of the studies is kept constant. The regression fit line is shown. The result is significant for maternal studies, with a negative correlation (r) of -0.62, and p value of 0.040. It is not significant for fetal studies.
