# Supplementary material for: Effect of interleukin-6 polymorphism on risk of preterm birth within population strata: a meta-analysis
Source: BMC Genet. 2013 Apr 25;14:30. doi: 10.1186/1471-2156-14-30 (PMC3639799; doi:10.1186/1471-2156-14-30)
Supplement: Additional file 4 — MOOSE checklist. [file 1471-2156-14-30-S4.pdf]

Additional file 4 – MOOSE checklist

This is the checklist of "meta-analyses of observational studies" (MOOSE). It showed how this study complied with the meta-analysis guideline.

**Criteria**		**In this paper:**
Reporting of background should include		
v	Problem definition	Association between preterm birth (PTB) and interleukin-6 (IL-6) protein is well established. Functional study between *IL6* gene and IL-6 protein has been done. But genetic study between PTB and *IL6* gene is inconsistent across different studies.
v	Hypothesis statement	SNP rs1800795 (*IL6* -174) derived genotype CC is protective against PTB
v	Description of study outcome(s)	Preterm Birth (PTB)
v	Type of exposure or intervention used	Genotype CC for SNP rs1800795 (*IL6*-174)
v	Type of study designs used	Include observational studies: case-control, cohort, or cross-sectional studies
v	Study population	Include all kinds of population studied, but we do subgroup analysis by populations
Reporting of search strategy should include		
v	Qualifications of searchers (eg, librarians and investigators)	Two investigators, WW and EC did the searches independently
v	Search strategy, including time period included in the synthesis and keywords	Use broad keywords with union of synonyms: "interleukin 6 polymorphism", or "interleukin 6 variant", or "interleukin 6 genotype", AND "preterm birth" OR "preterm delivery". Didn't restrict the time period to include all available studies. Didn't restrict the SNP because of different nomenclature but checked it manually.
v	Effort to include all available studies, including contact with authors	Use broad keywords with union of synonyms. Didn't restrict the time period to include all available studies. Didn't restrict the SNP because of different nomenclature but checked it manually. Authors were contacted for detail data (detailed in Supplementary Table 1)
v	Databases and registries searched	Pubmed
v	Search software used, name and version, including special features	No search software used
v	Use of hand searching	After broad search, we manually checked each paper to pull out rs1800795 under different names

v	List of citations located and those excluded, including justifications	Supplementary Table 1 listed all the citations and how they were included or excluded
v	Method of addressing articles published in languages other than English	One paper was excluded because of non-English
v	Method of handling abstracts and unpublished studies	Didn't include abstracts or unpublished studies
v	Description of any contact with authors	Supplementary Table 1 listed contacts with authors
Reporting of methods should include		
v	Description of relevance or appropriateness of studies assembled for assessing the hypothesis to be tested	Methods section and Supplementary Table 1 listed inclusion and exclusion criteria, and how they applied to each study.
v	Rationale for the selection and coding of data (eg, sound clinical principles or convenience)	Preterm birth is defined by gestational age. Genotype is defined by the sequence of the SNP. Both data are well-defined.
v	Documentation of how data were classified and coded (eg, multiple raters, blinding, and interrater reliability)	Two authors, WW and EC, independently searched and reviewed the articles. Mutual agreement was obtained before the final analysis.
v	Assessment of confounding (eg, comparability of cases and controls in studies where appropriate)	Hardy-Weinberg equilibrium(HWE) test was employed to excluded significantly deviated control group.
v	Assessment of study quality, including blinding of quality assessors; stratification or regression on possible predictors of study results	Each study was independently reviewed by two authors. Authors were contacted for missing data or stratifications.
v	Assessment of heterogeneity	Assessed by Cochrane's Q test of heterogeneity and I^2 statistic
v	Description of statistical methods (eg, complete description of fixed or random effects models, justification	Methods section described detail of statistical methods

	of whether the chosen models account for predictors of study results, dose-response models, or cumulative meta-analysis) in sufficient detail to be replicated	
v	Provision of appropriate tables and graphics	We provide a table for all papers included and excluded, forest plots to summarize meta-analysis results, tables for sensitivity tests, funnel plots for publication bias.
Reporting of results should include		
v	Graph summarizing individual study estimates and overall estimate	As in Figure 1 and 2 forest plots.
v	Table giving descriptive information for each study included	As in Table 1 and Supplementary Table 1
v	Results of sensitivity testing (eg, subgroup analysis)	As in Table 2 for sensitivity test
v	Indication of statistical uncertainty of findings	95% confidence intervals were reported with all point estimates
Reporting of discussion should include		
v	Quantitative assessment of bias (eg, publication bias)	As in Figure 3 and 4 funnel plots and Egger's test for publication bias, as in Table 2 for sensitivity test.
v	Justification for exclusion	Supplementary Table 1 listed these for each paper searched. Methods and Discussion also discussed the process
v	Assessment of quality of included studies	Supplementary Table 1 listed these for each paper searched. Authors were contacted if information is incomplete. Discussed about phenotype and population structure.
Reporting of conclusions should include		
v	Consideration of alternative explanations for observed results	Sensitivity test and publication bias were discussed in the discussion. Discussed about phenotype and population structure.
v	Generalization of the conclusions	As in Conclusions: rs1800795 CC genotype is protective against PTB in woman with European ancestry.
v	Guidelines for future research	As in Conclusions: The importance of population structure, and fetal studies in the future. Natural selection, phenotype clarification discussed in the Discussions.
v	Disclosure of funding source	N/A
